# Expression of antibody–drug conjugate targets in soft tissue sarcomas

**DOI:** 10.1016/j.esmoop.2025.105837

**Published:** 2025-10-04

**Authors:** F. Bertucci, P. Finetti, L. Mescam, A. Monneur, A. Frejafon, A. Le Cesne, I. Treilleux, A. Italiano, M. Brahmi, J.-Y. Blay, E. Mamessier

**Affiliations:** 1 Aix Marseille Univ, INSERM U1068, Institut Paoli-Calmettes, CRCM, “Predictive Oncology laboratory”, Label “Ligue contre le cancer”, Marseille, France; 2 Department of Medical Oncology, Institut Paoli-Calmettes, Marseille, France; 3 Department of Pathology, Institut Paoli-Calmettes, Marseille, France; 4 Department of Medical Oncology, Gustave Roussy, Villejuif, France; 5 Department of Pathology, Centre Léon Bérard, Lyon, France; 6 Department of Medical Oncology, Institut Bergonie, Bordeaux, France; 7 University of Bordeaux, Bordeaux, France; 8 Department of Medical Oncology, Centre Léon Bérard, Lyon, France

**Keywords:** antibody–drug conjugate, ADC, expression, prognosis, soft tissue sarcoma, survival

## Abstract

**Background:**

Soft tissue sarcomas (STSs) are aggressive and heterogeneous tumors with few efficient systemic therapies. Antibody–drug conjugates (ADCs) represent an emerging therapeutic option in oncology. Their efficacy is dependent on the expression of the ADC target on tumor cells. Very few data are available on ADC target expression in STSs.

**Materials and methods:**

We analyzed the mRNA expression of 62 targets and 60 genes potentially involved in resistance/response to ADC in 1664 clinical primary tumors, including 476 liposarcomas (LPSs) 341 leiomyosarcomas, 330 undifferentiated pleomorphic sarcomas, 286 gastrointestinal stromal tumors, 126 synovial sarcomas, and 105 myxofibrosarcomas. Tumor expression in each type was compared with expression in 7414 normal tissue samples. To confirm the results at the protein level, we applied immunohistochemistry (IHC) to four ADC targets in another series of STS samples.

**Results:**

Expression profiles of ADC targets were heterogeneous across and within all STS types. All types expressed multiple ADC targets. An overexpression rate of at least 25% of samples in at least one type was observed for 41 targets. The high target overexpression rate in some STS types suggested numerous new therapeutic opportunities not currently studied in clinical trials, such as PTK7 overexpressed in 81% of LPSs. In addition, co-expression of ADC-target pairs and of targets with signatures of vulnerability to immune checkpoint inhibitors, poly (ADP-ribose) polymerase (PARP) inhibitors, and cyclin-dependent kinase (CDK)4/6 inhibitors was evidenced in the different pathological types, suggesting opportunities for testing ADC-based combinations. Finally, we showed heterogeneous expression profiles of potential ADC resistance/response genes between and within STS types. IHC confirmed the mRNA results for the four tested targets.

**Conclusion:**

STSs express multiple target genes relevant for ADC treatment and expression varies between and within the pathological types. This comprehensive ADC target landscape, based on the largest molecular epidemiology study in STS, should help clinicians and drug developers for further evaluation of ADCs across STS types.

## Highlights

• Expression of 62 ADC targets and 60 “potential response” genes in 1664 clinical STS and 7414 normal tissue samples.
• Heterogeneous expression profiles of ADC targets between and within six major STS types.
• High overexpression rate of some ADC targets in some STS types.
• Co-expression of ADC-target pairs and of ADC targets with signatures of vulnerability to ICI or targeted therapies.
• Heterogeneous expression of potential ADC resistance/sensitivity genes across and within STS types.

## Introduction

Soft tissue sarcomas (STSs) are rare, aggressive, and heterogeneous tumors including over 100 different pathological and biological types.^1^ In early stages, >40% of operated patients experience metastatic relapse from which they will die. In patients with metastatic or unresectable disease, the treatment remains palliative, and the first-line systemic treatment remains anthracycline-based chemotherapy. The second-line therapies include chemotherapy (ifosfamide, dacarbazine, trabectedin, eribulin) and targeted therapy (pazopanib), but the results are disappointing. Improvement of systemic treatment is crucial.

Antibody–drug conjugates (ADCs) represent a major recent therapeutic advance in oncology.^2^ They combine a cytotoxic agent (payload) with a monoclonal antibody that is relatively specific for a tumor antigen via a combination molecule (linker). Their primary objective is to deliver the chemotherapy selectively to cells expressing the target antigen. ADC targets expressed on the surface of cancer cells do not need to be drivers of tumor growth to be meaningful, making ADCs potentially relevant for a wide range of tumors, notably sarcomas that lack clear oncogenic drivers. As of September 2024, seven ADCs were approved by the European Medicines Agency and/or the United States Food and Drug Administration (FDA) for solid cancers, and >150 novel ADCs are being tested in clinical trials. Efficacy of ADCs is well demonstrated in hematologic tumors and carcinomas, but their potential against sarcomas is still not completely explored.^3^^,^^4^ ADCs began to be tested in preclinical models and the promising results led some of them to enter clinical trials. Initial results suggested their validity as a promising therapy for patients with sarcoma. Examples include ADCs targeting AXL,^5^ LRRC15,^6^ ROR1 (NCT04441099 phase I-II), or ROR2 (NCT03504488 phase I).

Given their complex mechanism of action, the antitumor activity of ADCs depends on multiple tumor- and patient-intrinsic levels, including tumor expression of the target antigen, which overall correlates with the degree of activity,^2^ but also expression of other genes that influence internalization, linker cleavage, and sensitivity to the payload. High tumor expression of the target suggests the possibility to benefit from the corresponding ADC as an ‘agnostic therapy’, as illustrated by activity of trastuzumab deruxtecan in patients with human epidermal growth factor receptor 2 (HER2)-expressing tumors in the DESTINY-PanTumor02 trial.^7^

Very few data are available on the expression of ADC targets in sarcomas. Here, we examined the mRNA expression of ADC targets currently in preclinical and clinical development in a series of 1664 clinical STS samples and 7414 normal tissue samples. We also searched for correlations with disease-free survival and with therapeutic vulnerability to other systemic drugs being tested in combination with ADCs. We assessed expression of molecules possibly involved in response to ADCs. Finally, we applied immunohistochemistry (IHC) to validate our results for four ADC targets.

## Methods

### Soft tissue sarcoma samples and normal samples

We retrospectively gathered clinicopathological and gene expression data of clinical STS samples from 26 public data sets^8^, ^9^, ^10^, ^11^, ^12^, ^13^, ^14^, ^15^, ^16^, ^17^, ^18^, ^19^, ^20^, ^21^, ^22^, ^23^, ^24^, ^25^, ^26^, ^27^, ^28^, ^29^, ^30^, ^31^, ^32^, ^33^ (Supplementary Table S1, available at https://doi.org/10.1016/j.esmoop.2025.105837). A total of 2344 samples profiled using DNA microarrays or RNA sequencing (RNA-seq) were included. From this list, we selected the six pathological types with >100 cases, including 476 liposarcomas (LPS), 341 leiomyosarcomas (LMS), 330 undifferentiated pleomorphic sarcomas (UPS), 286 gastrointestinal stromal tumors (GIST), 126 synovial sarcomas (SVS), and 105 myxofibrosarcomas (MFS). The total number of selected STS samples was 1664. Gene expression data of 7414 normal tissue samples were collected from the Genotype-Tissue Expression (GTEx) database,^34^ representing 30 different healthy tissue types. We also analyzed cancer cell line data from the Dependency Map (DepMap) portal (https://depmap.org/portal) to compare the mass-spectrometry-based protein expression versus RNA-seq-based mRNA expression of all tested genes.

### ADC targets and predictive biomarkers

The list of analyzed ADC targets corresponded to the 54-target list recently developed by Bosi et al.^35^ that we completed by eight targets of ADCs under preclinical or clinical development in sarcomas. These latter were identified by searching in PubMed and ClinicalTrials.gov between July and September 2024 as follows. In PubMed, we used the following search terms: ‘antibody-drug conjugate’, ‘ADC’, ‘sarcoma’, and ‘GIST’, in various combinations, spelling variants and synonyms. The search was limited to articles published in English. Review articles^3^ served for cross-referencing. In ClinicalTrials.gov, we searched for clinical trials testing ADCs using the following search terms: [antibody-drug conjugate], [ADC], AND [sarcoma]. On September 2024, the trials concerned four ADC targets (AXL, LRRC15, ROR1, ROR2). For each ADC, the target name was collected. The selected final list included 62 unique targets. The list of biomarkers predictive for sensitivity or resistance to ADC corresponded to the 60-gene list previously reported.^35^

### Gene expression data analysis and statistical analyses

The pre-analytic processing first included normalization of each data set separately, by using Robust Multichip Average^36^ with the nonparametric quantile algorithm for the raw Affymetrix data and quantile normalization for the available processed non-Affymetrix microarray data. Normalization was done in R using Bioconductor and associated packages. Then, we mapped hybridization probes across the different technological platforms as reported.^37^ When multiple probes mapped to the same GeneID, we retained the one with the highest variance in each data set. We log_2_-transformed the already normalized RNA-seq data from the UCSC Xena transcriptome database, which includes The Cancer Genome Atlas (TCGA) tumor samples and the normal samples of the GTEx project. Next, the batch effects were corrected across the 26 studies using standardization on the 122 selected genes of interest (62 ADC targets and 60 response/resistance genes) and 150 genes used as controls for batch normalization (see Supplementary Methods, available at https://doi.org/10.1016/j.esmoop.2025.105837). Each study was heterogeneous, encompassing different types of STS. Such batch normalization was carried out in a stepwise manner, using LMS samples from TCGA as initial reference for average and standard deviation. For datasets that did not include LMS, standardization was carried out using a previously normalized dataset with the highest proportion of the same predominant non-LMS type as reference. Supplementary analyses, including hierarchical clustering and principal component analysis (PCA), were then applied to the prebatch normalization versus postbatch normalization data to show that batch effects had been effectively addressed and that the results provided were truly associated with the biology of the sarcomas under study as opposed to technical variation or batch effects associated with different datasets from different institutions/consortia (see Supplementary Methods, available at https://doi.org/10.1016/j.esmoop.2025.105837).

For each gene, the comparison of expression levels between STS samples and normal samples was done per STS type by computing both the percentage of samples in each type with an expression above the 80th percentile of the gene expression distribution across all normal samples, and the log_2_ fold change of STS samples/normal samples ratio. Hierarchical clustering was done using Euclidean distance and Ward linkage as parameters. The clinical endpoint of our prognostic analysis was the disease-free survival (DFS), calculated from the date of diagnosis until the date of metastatic relapse. The follow-up was measured from the date of diagnosis to the date of last news for event-free patients. Survival was calculated using the Kaplan–Meier method and curves were compared with the log-rank test. The cut-offs of the two strata were based on the median expression level of the gene in the population of interest. Univariate and multivariate analyses for DFS were done using Cox regression analysis (Wald test). Variables with a *P* value ≤0.05 in univariate analysis were included in the multivariate analysis. We also applied to each data set three gene expression signatures associated with vulnerability to different therapies: the tertiary lymphoid structure (TLS) signature^38^ associated with response to immune checkpoint inhibitors (ICIs), the homologous recombination deficiency (HRD) score associated with response to poly (ADP-ribose) polymerase inhibitors (PARPinh),^39^ and the E2Fregulon^40^ signature associated with response to cyclin-dependent kinase 4/6 inhibitors (CDK4/6inh). Analysis of correlations between continuous variables (gene expression levels and signature scores) was assessed by one-sided Pearson’s correlation coefficients (greater alternative) with *P* values adjusted for multiple comparisons using Bonferroni’s correction. Unless otherwise specified, the statistical tests were two-sided at the 5% level of significance. Statistical analysis was done using the survival package (version 2.43-3) in the R software (version 3.5.2; R Foundation for Statistical Computing, Vienna, Austria).

### Immunohistochemistry on tissue microarrays

In order to validate our transcriptomic results at the protein level, we carried out IHC on STS tissue microarrays (TMAs). Four TMAs were prepared at the ‘plate-forme d’Anatomopathologie Recherche de Lyon-Est’ from clinical tumor samples of patients with STS treated at the Centre Léon Bérard (Lyon, France). All samples were operative specimens, and none of them were present in our transcriptomic series. TMAs were prepared after careful selection of a representative tumor area for each sample by analysis of a hematoxylin–eosin-stained section of a donor block. For each tumor, three core cylinders (diameter of 1 mm) were punched from this area and deposited into a paraffin block using an arraying device.^41^ Sections of the resulting array block measuring 4 μm were made and transferred to glass slides before IHC analysis. A total of 94 STS were represented on four TMAs: TMA1 (24 LMS), TMA2 (22 dedifferentiated LPS: DD-LPS), TMA3 (24 UPS), and TMA4 (24 SVS). In addition, TMAs contained adequate negative and positive control tissues.

We selected four ADC targets from our series for which an antibody working well on formalin-fixed paraffin-embedded samples was commercially available and/or already available in our laboratory: PTK7, CD276 (B7H3), CD248 (endosialin), and HER2. The four antibodies were: anti-PTK7 (rabbit polyclonal, 1/50 dilution, R&D Systems, Minneapolis, USA), anti-CD276 (clone 1E7D1, 1/2500 dilution, Proteintech, Rosemont, USA), anti-CD248 (clone 1F9B4, 1/250 dilution, Proteintech, Rosemont, USA), and anti-HER2 (clone 4B5, ready to use, Roche, Rotkreuz, Switzerland). IHC was done on 4-μm sections in a BenchMark ULTRA™ stainer module (Roche, Rotkreuz, Switzerland) following manufacturer’s protocol. One STS expert pathologist (LM) carried out evaluation of the staining of tumor cells in a pathological type-blinded manner. CD276, CD248, PTK7, and HER2 showed membrane staining, and intensity of staining was scored from 0 (no staining) to 3 (strong and diffuse staining). For each tumor, the mean of intensities of the three cores was retained for analyses.

## Results

### Patient characteristics and ADC targets

Expression profiles of 1664 clinical samples of STS primary tumors were analyzed, including 476 LPS, 341 LMS, 330 UPS, 286 GIST, 126 SVS, and 105 MFS. Their characteristics are summarized in Table 1. The median patient age was 63 years (range 8-93 years), and 53% were males. The most frequent anatomical sites were extremities. The pathological tumor size on the surgical specimen was >5 cm in 73% of cases. Most non-GIST samples were grade 3, and 53% of GIST were low risk according to the Armed Forces Institute of Pathology (AFIP) system.^42^ DFS data were available for 773 patients: with a median follow-up of 32.5 months (range 0-222 months), 283 patients (37%) had a DFS event, and the 5-year DFS was 59% (95% confidence interval (CI) 55% to 63%].

**Table 1:** Clinicopathological characteristics of patients and sarcomas

Characteristics	All, *n* (%) (*n* = 1664)	LPS, *n* (%) (*n* = 476)	LMS, *n* (%) (*n* = 341)	UPS, *n* (%) (*n* = 330)	GIST, *n* (%) (*n* = 286)	SS, *n* (%) (*n* = 126)	MFS, *n* (%) (*n* = 105)
Median age, years (range)	63 (8-93)	61 (25-91)	61 (16-92)	66 (26-93)	59 (8-85)	37 (10-91)	65 (25-90)
Sex							
Female	412 (47)	55 (40)	116 (58)	81 (43)	100 (43)	26 (67)	34 (46)
Male	458 (53)	81 (60)	83 (42)	109 (57)	132 (57)	13 (33)	40 (54)
Tumor site							
Extremity	200 (42)	15 (14)	54 (32)	91 (64)	0 (0)	4 (50)	36 (77%)
Head and neck	7 (1)	1 (1)	1 (1)	3 (2)	0 (0)	1 (12)	1 (2)
Internal trunk	192 (40)	88 (79)	85 (51)	15 (10)	286 (100)	2 (25)	2 (4)
Superficial trunk	78 (16)	7 (6)	28 (17)	34 (24)	0 (0)	1 (12)	8 (17)
Pathological tumor size							
≤5 cm	105 (27)	5 (13)	33 (42)	35 (41)	48 (33)	2 (25)	13 (50)
>5 cm	278 (73)	33 (87)	46 (58)	51 (59)	98 (67)	6 (75)	13 (50)
Pathological tumor grade/AFIP classification							
I/low	NA	I: 54 (45)	I: 7 (5)	I: 0 (0)	Low: 85 (53)	I: 0 (0)	I: 1 (2)
Intermediate	NA				Intermediate: 31 (19)		
II-III/high	NA	II-III: 67 (55)	II-III: 145 (95)	II-III: 165 (100)	High: 45 (28)	II-III: 8 (100)	II-III: 40 (98)
Follow-up median, months (range)	32.5 (1-222)	29.8 (1-222)	21 (1-155)	34 (1-222)	49.5 (2-165)	22.4 (18-135)	29.5 (3-160)
Disease event	283 (37)	93 (36)	84 (56)	60 (30)	32 (26)	1 (20)	13 (33)
5-year DFS, % (95% confidence interval)	59 (55-63)	56 (49-64)	37 (29-48)	65 (58-73)	76 (67-85)	75 (43-100)	65 (51-84)

AFIP, Armed Forces Institute of Pathology; DFS, disease-free survival; GIST, gastrointestinal stromal tumor; LMS, leiomyosarcoma; LPS, synovial sarcoma; MFS, myxofibrosarcoma; NA, not applicable; SVS, synovial sarcoma; UPS, undifferentiated pleomorphic sarcoma.

The 62 selected ADC targets and the 60 biomarkers potentially associated with resistance/sensitivity to ADC are listed are listed in Supplementary Tables S2 and S3, available at https://doi.org/10.1016/j.esmoop.2025.105837 respectively. Before analyses, we measured the correlation between their mRNA and protein expression levels in the 363 DepMap cancer cell lines (Supplementary Tables S2 and S3, available at https://doi.org/10.1016/j.esmoop.2025.105837): expression levels for all 62 target genes were positively correlated, while 3 of the 60 resistance/response genes did not show significant positive correlation (*FAAP24*, *RPL28*, *SPCS1*) and were therefore excluded, leaving 57 genes for analysis.

### Expression of ADC targets

**Figure 1:**
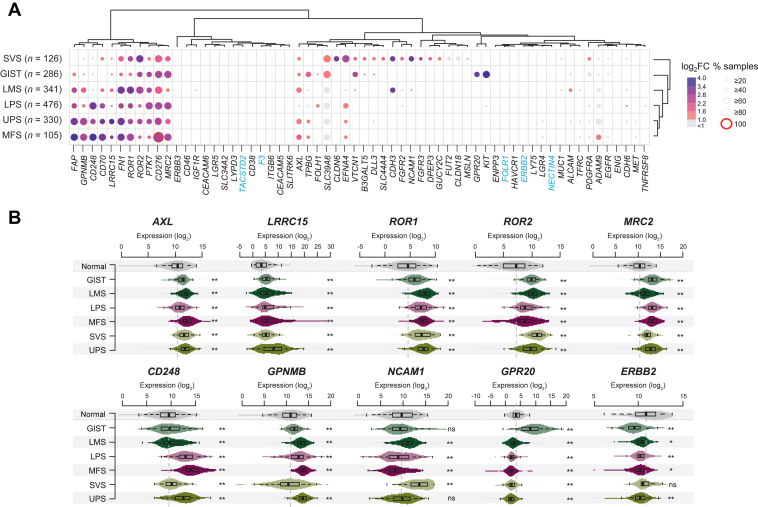
**Expression of antibody–drug conjugate (ADC) targets in soft tissue sarcoma (STS) and normal tissue samples.** (A) Expression of 62 ADC targets in each STS type compared with expression in all 7414 normal tissue samples. Each column represents one gene and each line represents one STS type. The dot size represents the percentage of STS samples in each type with expression superior to the 80th percentile of expression in normal samples, and the color code represents the tumor/normal tissue expression fold change (FC), as indicated in the scales (right). (B) Distribution of expression in STS types and normal tissues of nine targets from ADCs under clinical development in sarcomas (AXL, LRRC15, ROR1, ROR2, MRC2, CD248, GPNMB, NCAM, GPR20) and ERBB2. GIST, gastrointestinal stromal tumor; ns, not significant; LMS, leiomyosarcoma; LPS, liposarcoma; MFS, myxofibrosarcoma; SVS, synovial sarcoma; UPS, undifferentiated pleomorphic sarcoma. ∗*P* < 0.01; ∗∗*P* < 0.001.

Expression of ADC targets was heterogeneous among the different STS types (Supplementary Figure S1, available at https://doi.org/10.1016/j.esmoop.2025.105837). Two major tumor clusters were observed: the right cluster included most GIST, LMS, and SVS, while the left cluster included most LPS, UPS, and MFS. This imbalance was significant (*P* = 1.54E-131, Fisher’s exact test), indicating that GIST, LMS, and SVS have different expression profiles of ADC targets than other types. Figure 1A shows the expression of targets in STS types compared with expression in all normal tissue samples. All detailed values are listed in Supplementary Table S4, available at https://doi.org/10.1016/j.esmoop.2025.105837. All STS types overexpressed multiple targets at different levels. Supplementary Table S5, available at https://doi.org/10.1016/j.esmoop.2025.105837 shows the number of ADC targets overexpressed in at least 75%, 50%, 25%, and 10% of the samples. An overexpression rate of at least 75% of samples in at least one STS type was observed for 14 ADC targets, at least 50% for 26, at least 25% for 41, and at least 10% for 50. Analysis by STS type revealed no significant difference between the types in the number of ADC targets overexpressed (*P* > 0.05, chi-square test).

Overall, the most overexpressed ADC targets in all STS types were *CD276* (B7H3), *MRC2*, *PTK7*, *FN1*, *ROR1*, *ROR2*, and *LRRC15*, then *GPNMB*, *FAP*, *CD248*, *CD70*, then *AXL* and *EFNA4*. A few genes showed overexpression in only one pathological type, such as *KIT* in 80% of GIST or *NCAM1* in 66% of SVS. Other targets, such as those of ADCs currently marketed for carcinomas (*TACSTD2/*TROP2, *ERBB2/HER2*, *Nectin4*, *FOLR1*, *F3/*tissue factor), and *ERBB3*, *CD46*, *CEACAM5*, *CEACAM6*, *LRG5*, and *ENG* were overall not or rarely overexpressed in all STS types. Figure 1B shows the distribution of expression of nine targets of ADCs under clinical development in sarcomas: AXL, LRRC15, ROR1, ROR2, MRC2, CD248, GPNMB, NCAM, and GPR20. Most of them were overexpressed in each STS type compared with normal tissues. In contrast, *ERBB2/HER2* expression was lower in these STS types when compared with normal tissues.

### Prognostic value of ADC target expression

**Figure 2:**
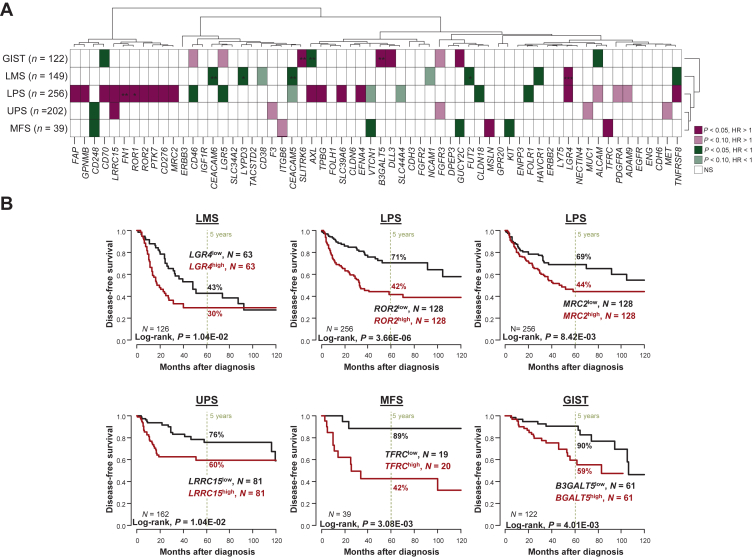
**Prognostic analysis of antibody–drug conjugate (ADC) target expression for disease-free survival in soft tissue sarcoma (STS).** (A) Results of Cox univariate analysis in each STS type. Each column represents one gene and each line represents one STS type. The genes are in the same order as Figure 1A. The color scale represents both the hazard ratio (HR; red: HR > 1, meaning poor prognosis; green: HR < 1, meaning good prognosis) and the degree of significance of the Wald test in univariate analysis (light: *P* < 0.10; dark: *P* < 0.05; white: not significant). No prognostic analysis was done in patients with synovial sarcoma (SVS) because of the low number of informative cases. No multivariate analysis was done in patients with undifferentiated pleomorphic sarcoma (UPS) and with myxofibrosarcoma (MFS). (B) Kaplan–Meier disease-free survival curves according to expression of six significant genes. GIST, gastrointestinal stromal tumor; HR, hazard ratio; LMS, leiomyosarcoma; LPS, liposarcoma; NS, not significant. The *P* values of multivariate analyses are indicated as follows: ∗*P* < 0.10; ∗∗*P* < 0.05; ∗∗∗*P* < 0.01.

We assessed the prognostic value of ADC target expression (continuous value) for DFS in each STS type (Figure 2A, Supplementary Table S6, available at https://doi.org/10.1016/j.esmoop.2025.105837). In patients with LMS [*N* = 149, 37% 5-year DFS (95% CI 29% to 48%)], *LGR4* expression showed unfavorable prognostic value, whereas expression of six targets (*TNFRSF8, FUT2, HAVCR1, CEACAM5, CEACAM6, LYPD3*) displayed favorable prognostic value. In multivariate analysis, three genes remained significant (*LGR4, CEACAM5, CEACAM6*). In the 256 patients with LPS [56% 5-year DFS (95% CI 49% to 64%)], 15 genes (*AXL, LRRC15, ROR1, ROR2, TNFRS8, PTK7, TPBG, FN1, LGR4, CD276, SLC39A6/LIV1, MRC2, FAP, CD70*, and *EFNA4*), including most targets strongly overexpressed in LPS, showed unfavorable prognostic value, whereas 4 (*CD46, CLND18, FOLR1, LGR5*) displayed a favorable prognostic value. In multivariate analysis, only *FN1* gene remained significant. In patients with UPS [*N* = 202, 65% 5-year DFS (95% CI 58% to 73%)], few genes displayed prognostic value: *LRRC15* showed an unfavorable prognostic value, whereas CD248 showed a favorable prognostic value. No multivariate analysis was done in UPS because of the low number of patients. In patients with MFS [*N* = 39, 65% 5-year DFS (95% CI 51% to 84%)], three targets (*MSLN, TFRC, ALCAM*) showed unfavorable prognostic value, and four (*VTCN1, GUCY2C, CD248, KIT*) displayed favorable prognostic value. No multivariate analysis was done in MFS because of the low number of patients. In patients with GIST [*N* = 122, 76% 5-year DFS (95% CI 67% to 85%)], four targets (*DLL3, GUCY2C, B3GALT5, SLITRK6*) showed unfavorable prognostic value, and three (*AXL, ALCAM, CD70*) displayed favorable prognostic value. Three genes (*SLITRK6, B3GALT5, AXL*) remained significant in multivariate analysis; Figure 2B shows the DFS curves according to expression of six significant genes. No prognostic analysis was done in patients with SVS because of the low number of informative cases.

### Co-expression of ADC targets and other therapeutic vulnerabilities

**Figure 3:**
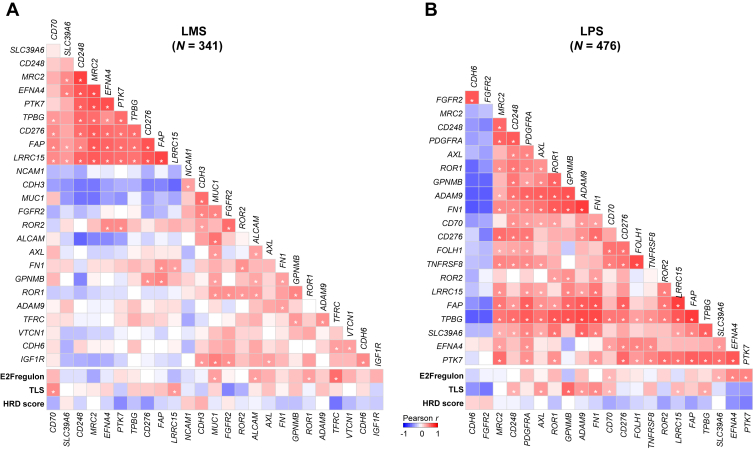
**Co-expression of antibody–drug conjugate (ADC) targets and signatures of therapeutic vulnerability in leiomyosarcoma (LMS) and liposarcoma (LPS).** Pair-wise correlation matrix of ADC target expression based on the Pearson correlation (*r*) in the LMS type (A) and the LPS type (B). The correlation is color-coded as indicated by the scale: red, positive correlation; blue, negative correlation; and darker colors indicate higher correlations. The significant correlations [one-sided Pearson correlation coefficients (greater alternative) with *P* values adjusted for multiple comparisons using Bonferroni’s correction] are indicated by a white star (q < 0.05). HRD, homologous recombination deficiency; TLS, tertiary lymphoid structure.

For each STS type, correlation of expression was measured between ADC-target pairs and between ADC targets and expression-based scores of vulnerability to ICI (TLS), PARPinh (HRD score), and CDK4/6inh (E2Fregulon). Analysis was restricted to ADC targets overexpressed in at least 20% of cases. The results are shown in Figure 3, Supplementary Figures S2 and S3, Supplementary Table S7, available at https://doi.org/10.1016/j.esmoop.2025.105837. The significance was assessed using Bonferroni’s correction. All correlations mentioned in the following text were significant but the strength of association ranged from weak (*r* = 0.2-0.39), to moderate (*r* = 0.4-0.59), strong (*r* = 0.6-0.79), or sometimes very strong (*r* = 0.8-1). For LMS, the strongest positive correlation in all ADC targets was between MRC2 and CD248 (*r* = 0.59), and between TFRC and E2Fregulon signature (*r* = 0.36). More positive correlations were significant for LPS. The strongest correlation was between FN1 and ADAM9 (*r* = 0.63), and between GPNMB and TLS signature (*r* = 0.38 with TLS). For UPS, the strongest correlation was between LLRC15 and FAP (*r* = 0.5), and between CD70 and the TLS signature (*r* = 0.23). For SVS, the strongest correlations were between GUCY2C and CLDN6 (*r* = 0.90), CD276 and TLS (*P* = 0.53), and B3GALT5 and TLS (*P* = 0.50). In GIST, the strongest correlations were between CD248 and PTK7 (*r* = 0.51), then between KIT and GPR20 (*r* = 0.48). Expression of GPR20 and AXL correlated with the HRD signature (*r* = 0.29), whereas expression of FAP and AXL correlated with the TLS signature (FAP: *r* = 0.37; AXL: *r* = 0.24), and FN1 expression with E2Fregulon signature (*r* = 0.28). For MFS, the strongest correlation was between LRRC15 and FAP (*r* = 0.48).

### Expression of genes associated with sensitivity or resistance to ADCs

**Figure 4:**
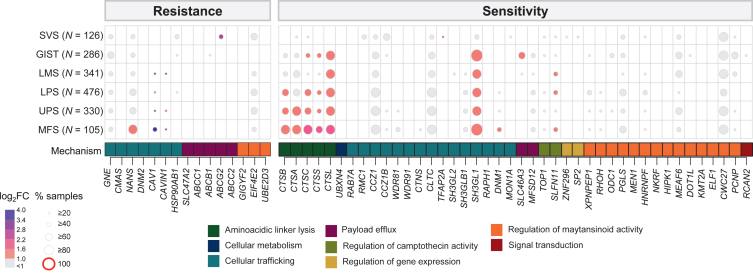
**Expression of genes associated with sensitivity or resistance to antibody–drug conjugate (ADCs) in soft tissue sarcoma (STS).** Expression of 57 genes involved in ADC resistance (15 genes) or sensitivity (42 genes) in each STS type compared with expression in all 7414 normal tissue samples. Each column represents one gene and each line represents one STS type. The dot size represents the percentage of STS samples in each type with expression superior to the 80th percentile of expression in normal samples and the color code represents the tumor/normal tissue expression fold change (FC), as indicated in the scales (left). The genes are ordered by broad mechanism of action (indicated between the matrix and the gene symbols and the color scale below). GIST, gastrointestinal stromal tumor; LMS, leiomyosarcoma; LPS, synovial sarcoma; MFS, myxofibrosarcoma; SVS, synovial sarcoma; UPS, undifferentiated pleomorphic sarcoma.

We analyzed the expression of 57 genes potentially involved in ADC response.^35^ The results are detailed in Supplementary Table S8, available at https://doi.org/10.1016/j.esmoop.2025.105837. As with ADC targets, heterogeneous expression levels within STS types were observed (Figure 4). Overall, the expression profiles of these genes appeared to be relatively similar among all types of sarcoma except SVS. Among the 15 ‘resistance genes’, those involved in drug efflux (ABC transporters, *SLC47A2*) were not or rarely overexpressed in all pathological types. Also, none of the genes associated with resistance to maytansinoids were overexpressed, except *EIF4E2*, highly expressed in all non-GIST types. In contrast, several ‘resistance genes’ involved in cellular trafficking, such as GNE, were overexpressed in at least 20% of samples in all types.

Regarding the 42 ‘sensitivity genes’, cathepsin genes, encoding peptidases involved in the lysis of aminoacidic linkers, notably Cathepsin L (CTSL), were overexpressed in all non-SVS types. Their expression was highest in MFS. Among the ‘sensitivity genes’ involved in cellular trafficking, *SH3GL1* was strongly overexpressed in all non-SVS types. *TOP1* and *SLFN11*, whose expression is associated with sensitivity to TOP1 inhibitors, were overexpressed respectively in >20% of non-LMS types, in >40% of LMS, LPS, and MFS, and in >20% of other types. Of the predictors of sensitivity to maytansinoids, some showed no overexpression in all STS types (*MEN1, NKRF, HIPK1, KMT2A, ELF1*), while others showed overexpression in all types (*PGLS, MEAF6, CWC27*).

### Expression of ADC targets in LPS subtypes

To further assess heterogeneity within the STS types, notably LPS, we analyzed expression of ADC targets across the three major pathological subtypes of LPS: well differentiated (WD)/DD (*N* = 284), myxoid (*N* = 111), and pleomorphic (*N* = 81). Most of the targets showed similar expression profiles in the WD/DD and pleomorphic subtypes (Supplementary Figure S4, Supplementary Table S4, available at https://doi.org/10.1016/j.esmoop.2025.105837). Six targets were overexpressed in at least 80% of WD/DD-LPS (CD276, MRC2, CD248, SLC39A6, PTK7, FN1), while three were overexpressed in at least 80% of pleomorphic LPS (CD276, CD248, FN1). Myxoid LPS display more differences with both WD/DD and pleomorphic LPS. For example, FGFR2, CDH6, and IGF1R were overexpressed in 74%, 85%, and 41% of myxoid LPS, respectively, versus only 12%, 25%, and 7% of WD/DD-LPS and 2%, 23%, and 2% of pleomorphic LPS. All LPS subtypes overexpressed multiple targets at different levels. Supplementary Table S5, available at https://doi.org/10.1016/j.esmoop.2025.105837 showed that nearly twofold more ADC targets were overexpressed in at least 50% of samples in WD/DD-LPS and pleomorphic LPS than in myxoid LPS, even if the difference was not significant (*P* > 0.05, chi-square test). Among the targets of ADCs currently marketed for carcinomas (*TACSTD2/*TROP2, *ERBB2/HER2*, *Nectin4*, *FOLR1*, *F3/*tissue factor), none of them were overexpressed in at least 10% of samples in each LPS subtype, except FOLR1 found as overexpressed in 15% of myxoid LPS and F3 overexpressed in 11% of WD/DD-LPS samples.

### Validation of ADC target expression at the protein level

**Figure 5:**
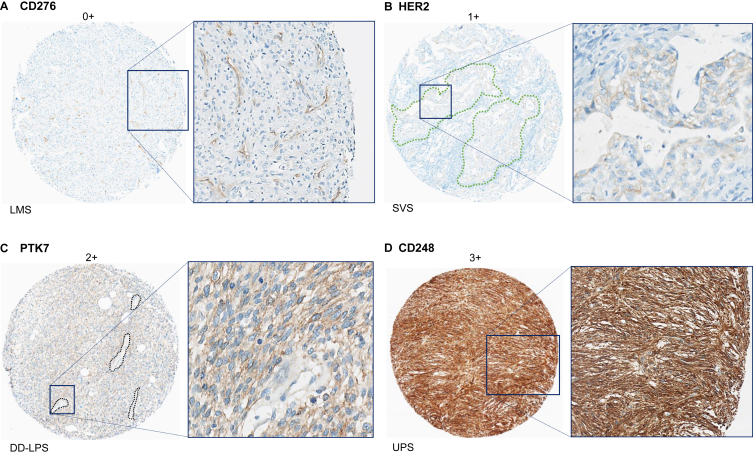
**Examples of protein expression of four antibody–drug conjugate (ADC) targets in soft tissue sarcoma.** Images were scanned with an objective ×40. One tumor core is shown per antibody (left image) with a zoom of a specific zone (defined by a blue square on the tumor core) on the image to the right. (A) Example of CD276 null expression on tumor cells in a leiomyosarcoma (LMS) sample. Positive staining can, however, be seen on the cells forming the blood vessels. (B) Human epidermal growth factor receptor 2 (HER2) expression, scored with a grade 1 intensity, can be seen in epithelioid pseudoglandular foci (two examples of foci have been delineated with green dashed lines) in a synovial sarcoma (SVS) sample. As expected, the stromal cells are negative for HER2. (C) PTK7, scored here with a grade 2 intensity, shows an expression in tumor cells of a dedifferentiated synovial sarcoma (DD-LPS) sample. Areas with vessels showed low/null staining (black dashed line). (D) Example of CD248 protein expression, scored here with a grade 3 intensity, in an undifferentiated pleomorphic sarcoma (UPS) sample. All the cells composing the tumor show a strong staining.

Expression of four ADC targets was assessed using IHC in an independent series of 94 STS: 24 LMS, 22 DD-LPS, 24 UPS, and 24 SVS. Examples of staining are shown in Figure 5. Overall, the most frequently expressed targets in the whole STS series were CD276 (B7H3) expressed in 91% of cases, followed by PTK7 expressed in 65%, then CD248 in 35%, and HER2 in 3%. These positivity rates (Supplementary Table S9, available at https://doi.org/10.1016/j.esmoop.2025.105837) were very close to those observed at the mRNA level in the series including all 341 LMS, 330 UPS, and 126 SVS, and all 218 DD-LPD (we excluded here the other LPS subtypes in order to be comparable to the IHC series).

CD276 (B7H3) protein was expressed in 91% of STS, a rate close to the 97% rate found at the mRNA level. Analysis per pathological type also confirmed the mRNA results. For example, 92% of SVS expressed CD276 at the protein level versus 93% at the mRNA level. Expression of PTK7 protein was observed in 65% of STS, thus confirming the 68% rate observed at the mRNA level. Analysis per pathological type also confirmed our results in DD-LPS (86% at both protein and mRNA levels) and UPS (63% versus 50%, respectively), In SVS, 100% of SVS expressed the PTK7 protein, including 39% with strong staining intensity. CD248 protein was expressed in 35% of STS. The highest expression rate was found in DD-LPS (62%), followed by UPS (50%), as observed at the mRNA level. (83% and 72% respectively). The expression rate in SVS was similar at protein and mRNA levels (25%). HER2 protein was never expressed in LMS, DD-LPS, and UPS, whereas positivity was observed in 3 out of 24 SVS (17%), in agreement with our mRNA results. Of note, the 4 HER2-positive cases corresponded to biphasic SVS, which displayed a 66% positivity rate (4 out of 6), whereas all 16 monophasic spindle cell SVS showed negative staining.

## Discussion

Because of the increasing importance of ADCs in oncology and the lack of data on target expression in STS, we analyzed mRNA expression of 62 ADC targets and 57 other proteins potentially involved in ADC efficacy in a series of 1664 previously untreated operated STS samples representing six major pathological types and 7414 normal tissue samples. To our knowledge, this is the second molecular epidemiology study on expression of ADC targets in STS and by far the largest one in terms of the number of samples and targets analyzed.

Our analysis was based on mRNA expression. IHC would have allowed the investigation of the protein expression of targets. However, a large-scale IHC analysis for the 119 molecules analyzed here would be time-consuming and demand extensive resources. This is particularly true in STS, the great diversity of which complicates the task of a large screen in specific pathological types. Transcriptome data allowed us to avoid such limitations. Our preliminary analysis of >350 cancer cell lines revealed a strong correlation between mRNA and protein expression levels, allowing us to base our study on gene expression data.^43^, ^44^, ^45^, ^46^, ^47^, ^48^ This approach provided the opportunity to work with a large number of targets and samples, to search for associations with relevant multigene expression signatures, and to compare expression levels between tumors and pooled normal tissues. Two similar studies evaluated gene expression of multiple ADC targets in many cancer types, but none was specific to STS.^35^^,^^49^ In the sole study dedicated to STS,^50^ the authors compared expression of 8 ADC targets in ∼250 TCGA STS samples between LMS, LPS, UPS, SVS, MFS, and malignant peripheral nerve sheath tumor. No prognostic analysis nor analyses of co-expression or of expression of sensitivity/resistance genes were carried out.

We found significant differences in expression profiles of the 62 ADC targets across STS types. For each target, the rate of tumor overexpression allowed the identification of the most appealing STS type(s) for the corresponding ADC. As expected, *ERBB2*, *TACSTD2/*TROP2, *Nectin4*, *FOLR1*, and *F3/*tissue factor, which encode targets of ADCs marketed for carcinomas, were not overexpressed in STS types, supporting the validity of our methodology. We confirmed this result at the protein level for HER2, which was found as expressed in only 3% of all STS samples, in 0% of LMS, DD-LPS, and UPS, but in 17% of SVS. This latter corresponded to four out of six biphasic SVS that showed a moderate positivity (intensity: 1) in 10% to 70% of tumor cells (HER2-low), whereas all monophasic spindle cell SVS were negative. Clearly, HER2 IHC analysis is warranted in a larger series of SVS, including monophasic and biphasic cases, to define the percentage of HER2-low tumors that might be offered trastuzumab deruxtecan. We also confirmed high expression of some ADC targets in all STS types. For example, *CD276* was one of the most overexpressed genes in all STS types, and our IHC analysis confirmed these results. This immune checkpoint is frequently overexpressed in sarcomas^51^^,^^52^ and the anti-B7H3 ADC, m276-SL-PBD, showed strong activity against preclinical models.^52^ To our knowledge, only one study reported IHC results for B7H3 in 153 STS:^53^ expression was positive in ∼97% of samples and was high (intensity = 3) in ∼69%. These rates were similar to what we found here at both mRNA and protein levels. Similarly, *MRC2* (uPARAP/Endo180) was overexpressed in all sarcoma types, in >40% of LMS to 87% of LPS, as reported at the protein level in a 625-case series.^54^ Today, uPARAP is an attractive emerging target for the development ADCs in STS.^55^ However, the high expression of these two genes was not known in GIST for example. Other examples of frequently overexpressed targets included targets of ADC being tested in sarcomas, such as ROR1, ROR2, LRRC15, and AXL. However, their overexpression rate differed depending on the STS type. *LRRC15* was overexpressed in 36% to 45% of non-UPS types but in 65% of UPS, as reported in an IHC study of 711 cases.^56^ In the 10 UPS patients enrolled in the phase I trial (NCT02565758) testing the ABBV-085 ADC,^6^ the objective response rate was 20%. *AXL* overexpression rate ranged from 30% in LPS to 74% in GIST, that of *ROR1* from 39% in GIST to 83% in LMS and MFS, and that of *ROR2* from 41% in MFS to 90% in SVS. This heterogeneity in expression across STS types confirms that the evaluation of personalized therapies such as ADCs is a major challenge that must be documented. Such heterogeneity was also observed across the three LPS subtypes (WD/DD, pleomorphic, myxoid), and more differences were observed between myxoid versus WD/DD and pleomorphic subtypes than between WD/DD versus pleomorphic subtypes. Some targets were found to be overexpressed in only one STS type. Examples include KIT and GPR20 in GIST, in agreement with previous findings.^57^ Targeting GPR20 with the DS-6157a ADC results in tumor shrinkage in *KIT/PDGFRA* wild-type patients with advanced GIST.^58^ Importantly, the frequent and previously unknown overexpression of targets in some STS types suggests numerous new therapeutic opportunities that are not currently being investigated in clinical trials. Examples include *PTK7* overexpressed in 81% of LPS, *CDH3* in 67% of LMS and 66% of SVS, *VTCN1* in 73% of GIST, *NCAM1* in 66% of SVS, and *ADAM9* in 72% of MF and 61% of UPS. With 65% of STS samples showing positive PTK7 staining, IHC analysis confirmed our mRNA results. Of note, 14% displayed a strong staining. No data are available in the literature on PTK7 expression in STS. Our results observed on 1664 samples (mRNA) and 94 samples (protein) suggest great potential of anti-PTK7 ADCs in STS, notably in LPS and SVS. Regarding CD248, expression was higher at RNA and protein levels in DD-LPS and UPS than in better differentiated STS, as reported.^59^ Higher CD248 positivity rates observed at the mRNA versus protein levels may be due to post-transcriptional and/or post-traductional regulations, but also to expression of endosialin by both tumor cells and stromal cells of a range of STS.

We also assessed the prognostic value of ADC target expression. Even though the main mechanism of action of ADCs is the targeted delivery of chemotherapy within cancer cells, it is interesting to determine the prognosis of tumors overexpressing the target. Furthermore, in a few cases, the target may be the driver of the cancer cell, and its inhibition may lead to some anticancer effect (e.g. anti-HER2 ADCs in breast cancer). In our analysis, no target displayed a significant prognostic impact in all STS types. Some targets showed a similar prognostic value across several types (e.g. favorable impact of *CD248* expression in UPS and MFS, unfavorable impact of *LRRC15* in LPS and UPS). Expression of other targets, such as *CD70* or *AXL* in LPS and GIST, showed opposite prognostic value across different types, further reflecting STS heterogeneity and the need to perform prognostic analyses per pathological type. Our results confirmed previous data for some genes such as *CD276*^60^ or LRRC15,^56^ but for most significant genes, the prognostic value has never been tested in STS and in specific types.

The combination of ADC with other therapies is being explored in oncology.^61^^,^^62^ Expression of multiple ADC targets in the same tumor suggests the possibility of increasing anticancer effects by combining different ADCs or using bispecific ADCs. To date, only one clinical trial testing combinations of ADCs was reported.^63^ Twenty-four patients with refractory metastatic urothelial carcinoma received the enfortumab–vedotin/sacituzumab–govitecan combination: an impressive 70% objective response rate was reported. We recently proposed the co-expression of antigen targets as a rationale for such activity.^64^ We identified significant co-expression of multiple different ADC targets, variable according to STS types, suggesting opportunities to design clinical trials testing ADC combinations. These significant positive correlations showed a wide range of values, representing a strength of association ranging from weak (*r* = 0.2-0.39), to moderate (*r* = 0.4-0.59), strong (*r* = 0.6-0.79), or sometimes very strong (*r* = 0.8-1).^64^ Examples of combinations might include anti-MRC2 and anti-CD248 in LMS (*r* = 0.59), anti-FN1 and anti-ADAM9 in LPS (*r* = 0.63), anti-LLRC15 and anti-FAP in UPS (*r* = 0.5), anti-GUCY2C and anti-CLDN6 (*r* = 0.90) or anti-ROR1 and anti-ROR2 in SVS (*r* = 0.4), or anti-CD248 and anti-PTK7 in GIST (*r* = 0.51). Similarly, the co-expression of ADC with biomarkers predictive for response to other therapies may suggest relevant drug combinations. Regarding ICI in combination with ADCs, clinical trials are ongoing with promising results. Yet, in April 2023, the FDA approved the breakthrough for enfortumab vedotin (antinectin4 ADC) and pembrolizumab in cisplatin-ineligible advanced urothelial cancer. Even if the strength of association is weak or moderate, the positive correlations that we observed between the expression of an ADC target and the TLS signature in a given STS type suggest that the latter is a particularly relevant candidate for the ADC–ICI combination despite the limited activity of single-agent ICI in STS. Examples of ADCs include anti-LRRC15 in LMS (*r* = 0.23), UPS (*r* = 0.20), and SVS (*r* = 0.52); anti-GPNMB in LPS (*r* = 0.38); anti-CD276 in SVS (*P* = 0.53); or anti-CD70 in UPS (*r* = 0.23). Combining agents targeting the DNA damage response, such as PARPinh, with ADCs carrying DNA-damaging agents is a promising strategy for genetically unstable tumors.^62^ Even if the strength of association is weak (*r* = 0.29), the co-expression of GPR20 with HRD signature suggests that the combination of anti-GPR20 ADC with PARPinh should be evaluated in GIST.

The response or resistance to ADC depends on many tumor parameters, including the ADC target expression, but also other biological processes related to the target antigen, to ADC trafficking/processing within the cell, and to the payload. To date, these resistance mechanisms are beginning to be understood,^65^ but no biomarker is used in clinics. However, it is crucial to identify the types and proportions of STS that are potentially candidates to a given ADC. In a first exploratory approach, we analyzed the expression of 57 genes encoding potential actors of response/resistance to ADCs. Overall, expression profiles were heterogeneous within all STS types, but appeared relatively similar between all but SVS types. Among the ‘resistance genes’, those involved in drug efflux^66^ were not or rarely overexpressed, likely in part because no previous systemic therapy was administered before surgery. ADCs currently tested in clinical trials in STS contain microtubule-targeting agents as payload.^5^^,^^6^ Among the genes associated with resistance to maytansinoids, *EIF4E2* was overexpressed in 54% to 69% of all non-SVS types. Several ‘resistance genes’ involved in cellular trafficking, such as *GNE*, were overexpressed in at least 20% of samples in all types of STS. Regarding the ‘sensitivity genes’, cathepsin genes were frequently overexpressed in MFS and UPS as reported,^67^ but not in SVS. These proteases are activated in the lysosomes and involved in the cleavage of ADC linkers. Of the genes involved in cellular trafficking, *SH3GL1* was strongly overexpressed in all but SVS types. It encodes Endophilin A2, which promotes HER2 internalization: its silencing in HER2-positive cancer cells impaired HER2 internalization in response to trastuzumab, and resulted in a reduced cytotoxicity response in cells treated with T-DM1 ADC.^68^ Numerous TOP1-inhibitor-based ADCs are in clinical trials in numerous cancer types and will compete with monomethyl auristatin E (MMAE)-based ADCs in STS.^4^ *TOP1* and *SLFN11* were overexpressed respectively in >20% of all non-LMS types, in >40% of LMS, LPS, and MFS, and >20% of other types, making these tumors potential candidates for TOP1-inhibitor-based ADCs.

### Conclusion

We showed (i) heterogeneous expression profiles of ADC targets between and within six major STS types, (ii) a high overexpression rate of some ADC targets in some types, (iii) a prognostic value of overexpression of some targets in some STS types, (iv) a co-expression of ADC-target pairs and of ADC targets with signatures of vulnerability to systemic therapies, and (v) heterogeneous expression of potential ADC resistance/sensitivity genes. The strengths of our study are the size of the series (with 1664 STS, it is the largest series of STS reported to date), the high number of ADC targets analyzed, and analysis per STS type taking into account the actual heterogeneity of disease. Our study has some limitations. Firstly, it is retrospective and multicentric, thus inherently associated with potential biases. Secondly, the results observed at the mRNA level may not fully translate to protein expression levels. However, we limited our analysis to genes showing significant protein/mRNA correlation in a large panel of cancer cell lines, and our IHC analysis applied to four ADC targets in an independent series of STS samples confirmed the results observed at the mRNA level. Thirdly, gene expression levels were defined on the bulk tumor samples, impeding defining expression on cancer and/or immune cells. Fourthly, the correlation between ADC target expression and ADC activity is not clear for all ADCs. Fifthly, our analysis of potential sensitivity/resistance genes was based on a nonexhaustive list relative to the numerous unknowns in the field. Of course, analysis of larger retrospective then prospective series, at the protein level, is warranted. But yet, our results suggest many potentially effective cell surface antigens against which ADCs can be directed in different STS types. Expression of these targets may serve as a potential marker for patient selection in early clinical trials.

## Supplementary data

Supplementary Figures and Tables Legends

Supplementary Methods Revised
