# Supplementary material for: Expression of antibody–drug conjugate targets in soft tissue sarcomas
Source: ESMO Open. 2025 Oct 4;10(10):105837. doi: 10.1016/j.esmoop.2025.105837 (PMC12528890; doi:10.1016/j.esmoop.2025.105837)
Supplement: Supplementary Figure 1 [file mmc1.pptx]

## Slide 1
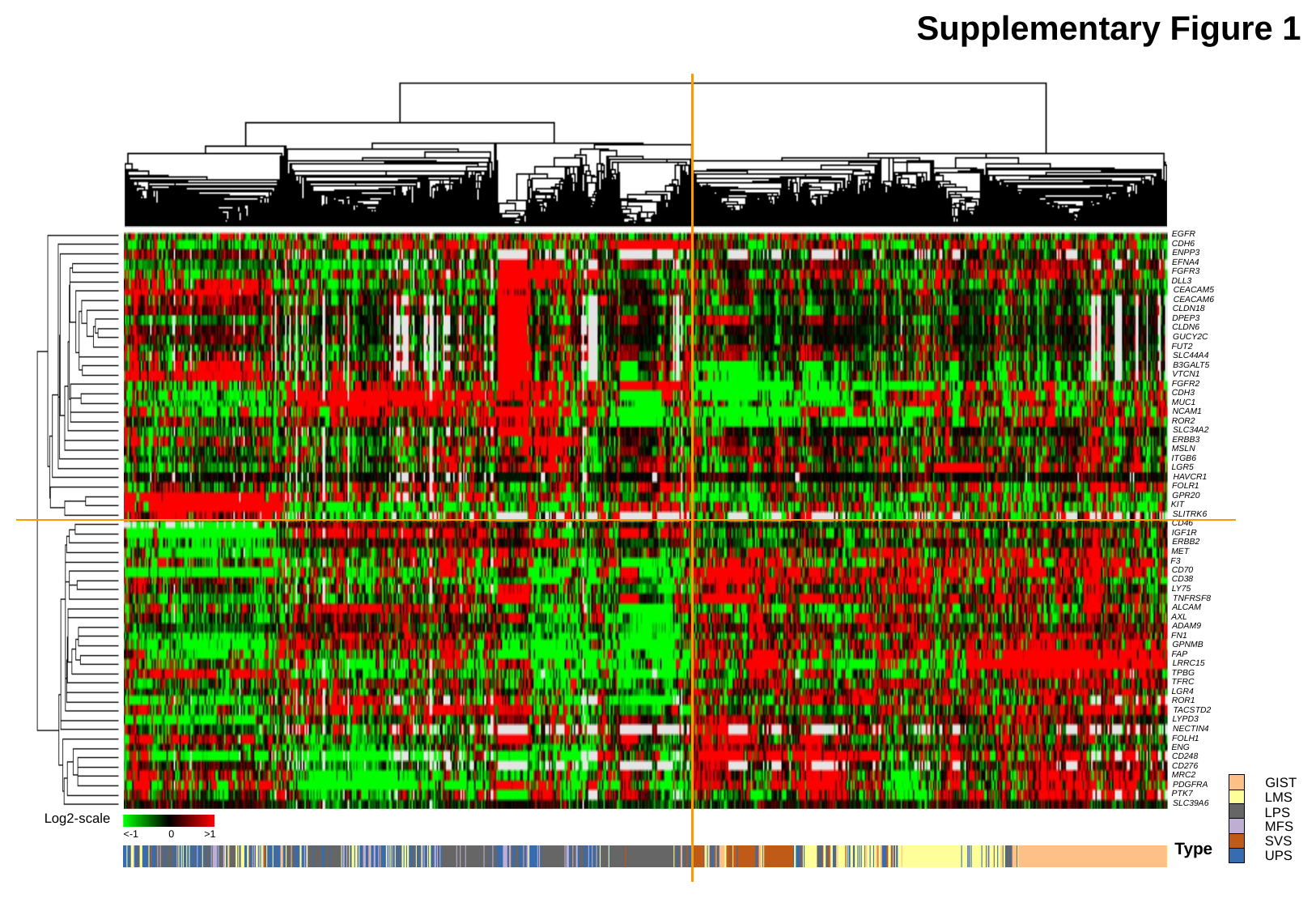

Supplementary Figure 1
EGFR
CDH6
ENPP3
EFNA4
FGFR3
DLL3
CEACAM5
CEACAM6
CLDN18
DPEP3
CLDN6
GUCY2C
FUT2
SLC44A4
B3GALT5
VTCN1
FGFR2
CDH3
MUC1
NCAM1
ROR2
SLC34A2
ERBB3
MSLN
ITGB6
LGR5
HAVCR1
FOLR1
GPR20
KIT
SLITRK6
CD46
IGF1R
ERBB2
MET
F3
CD70
CD38
LY75
TNFRSF8
ALCAM
AXL
ADAM9
FN1
GPNMB
FAP
LRRC15
TPBG
TFRC
LGR4
ROR1
TACSTD2
LYPD3
NECTIN4
FOLH1
ENG
CD248
CD276
MRC2
PDGFRA
PTK7
SLC39A6
GIST
LMS
LPS
MFS
SVS
UPS
Log2-scale
0
<-1
>1
Type
