# Supplementary material for: Expression of antibody–drug conjugate targets in soft tissue sarcomas
Source: ESMO Open. 2025 Oct 4;10(10):105837. doi: 10.1016/j.esmoop.2025.105837 (PMC12528890; doi:10.1016/j.esmoop.2025.105837)
Supplement: Supplementary Figure 2 [file mmc2.pptx]

## Slide 1
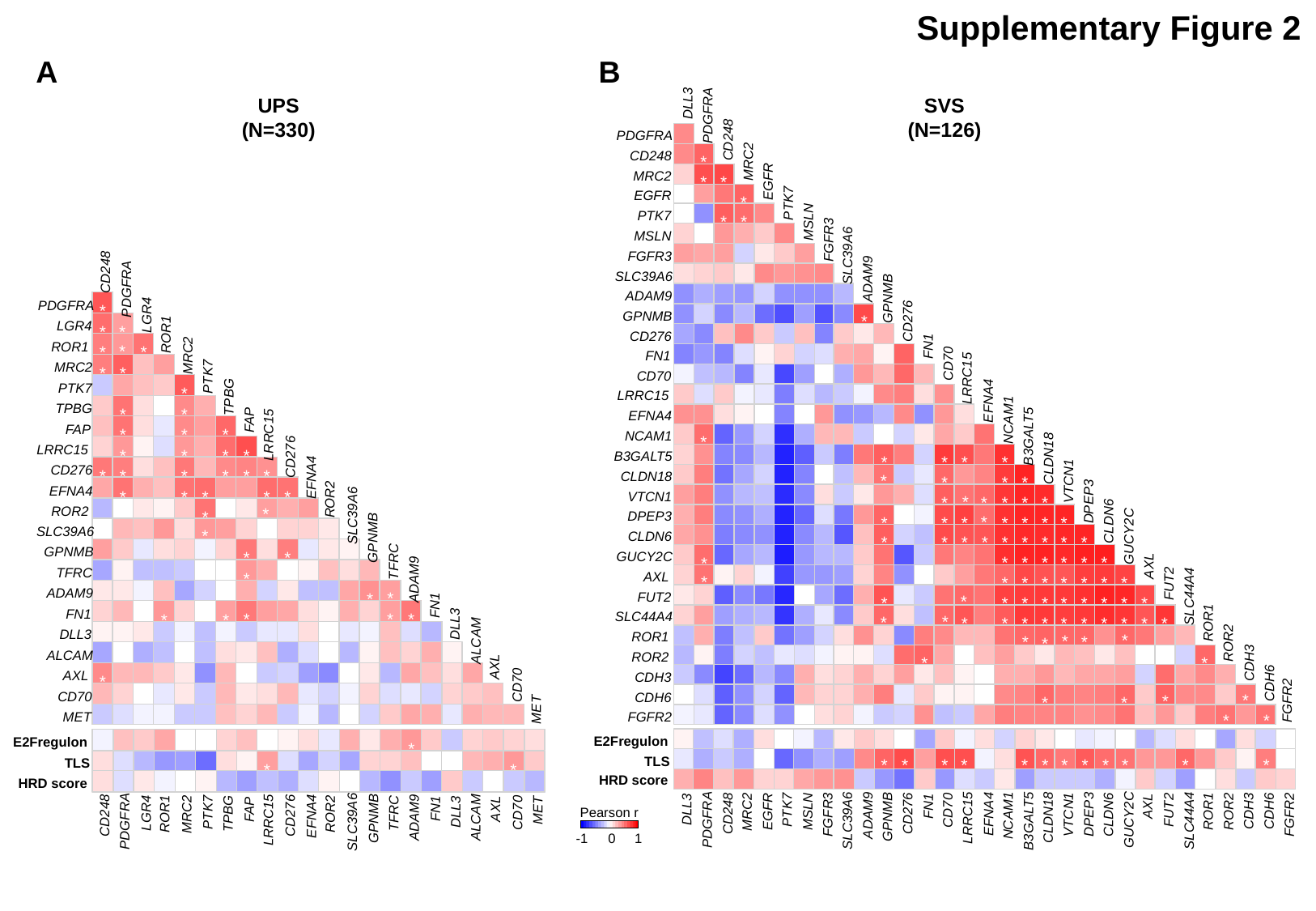

Supplementary Figure 2
A
B
UPS
(N=330)
SVS
(N=126)
DLL3
PDGFRA
CD248
MRC2
EGFR
PTK7
MSLN
FGFR3
SLC39A6
ADAM9
GPNMB
CD276
FN1
CD70
LRRC15
EFNA4
NCAM1
B3GALT5
CLDN18
VTCN1
DPEP3
CLDN6
GUCY2C
AXL
FUT2
SLC44A4
ROR1
ROR2
CDH3
CDH6
PDGFRA
CD248
MRC2
EGFR
PTK7
MSLN
FGFR3
SLC39A6
ADAM9
GPNMB
CD276
FN1
CD70
LRRC15
EFNA4
NCAM1
B3GALT5
CLDN18
VTCN1
DPEP3
CLDN6
GUCY2C
AXL
FUT2
SLC44A4
ROR1
ROR2
CDH3
CDH6
FGFR2
FGFR2
*
*
*
*
*
*
*
*
*
*
*
*
*
*
*
*
*
*
*
*
*
*
*
*
*
*
*
*
*
*
*
*
*
*
*
*
*
*
*
*
*
*
*
*
*
*
*
*
*
*
*
*
*
*
*
*
*
*
*
*
*
*
*
*
*
*
*
*
*
*
*
*
*
*
*
*
*
*
*
*
*
*
*
*
*
*
*
*
*
E2Fregulon
TLS
*
*
*
*
*
*
*
*
*
*
*
*
HRD score
AXL
FN1
DLL3
FUT2
PTK7
CD70
ROR1
ROR2
CDH3
CDH6
EGFR
MSLN
MRC2
CD248
CD276
EFNA4
VTCN1
CLDN6
FGFR2
FGFR3
DPEP3
ADAM9
NCAM1
GPNMB
LRRC15
CLDN18
PDGFRA
GUCY2C
SLC39A6
SLC44A4
B3GALT5
CD248
PDGFRA
LGR4
ROR1
MRC2
PTK7
TPBG
FAP
LRRC15
CD276
EFNA4
ROR2
SLC39A6
GPNMB
TFRC
ADAM9
FN1
DLL3
ALCAM
AXL
CD70
PDGFRA
LGR4
ROR1
MRC2
PTK7
TPBG
FAP
LRRC15
CD276
EFNA4
ROR2
SLC39A6
GPNMB
TFRC
ADAM9
FN1
DLL3
ALCAM
AXL
CD70
MET
MET
*
*
*
*
*
*
*
*
*
*
*
*
*
*
*
*
*
*
*
*
*
*
*
*
*
*
*
*
*
*
*
*
*
*
*
*
*
*
*
*
*
*
*
E2Fregulon
*
TLS
*
*
HRD score
AXL
FN1
FAP
MET
DLL3
PTK7
ROR1
ROR2
CD70
LGR4
TFRC
TPBG
MRC2
CD248
CD276
EFNA4
ADAM9
ALCAM
LRRC15
GPNMB
PDGFRA
SLC39A6
Pearson r
-1
0
1
