# Supplementary material for: Expression of antibody–drug conjugate targets in soft tissue sarcomas
Source: ESMO Open. 2025 Oct 4;10(10):105837. doi: 10.1016/j.esmoop.2025.105837 (PMC12528890; doi:10.1016/j.esmoop.2025.105837)
Supplement: Supplementary Figure 3 [file mmc3.pptx]

## Slide 1
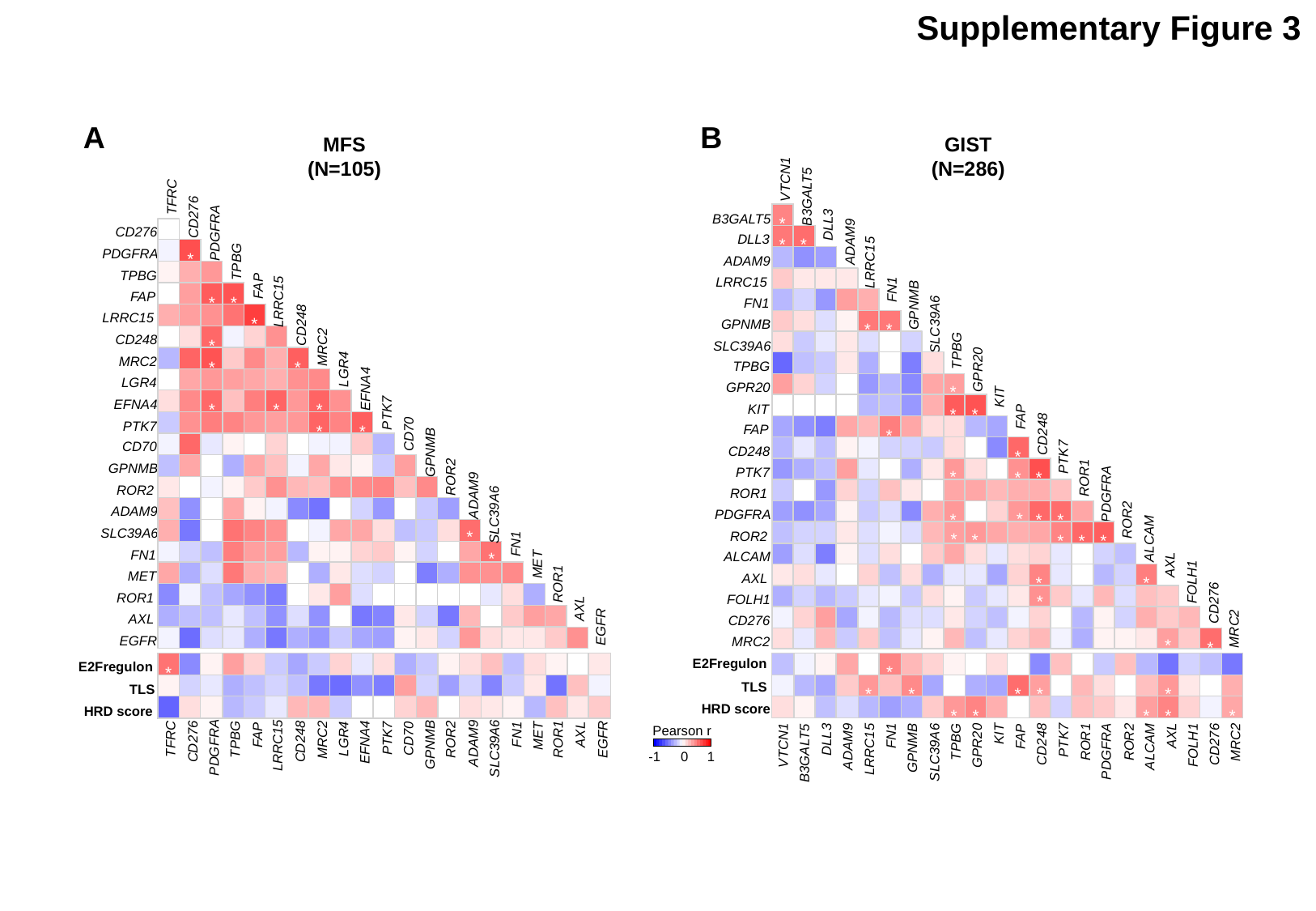

Supplementary Figure 3
A
B
MFS
(N=105)
GIST
(N=286)
VTCN1
B3GALT5
DLL3
ADAM9
LRRC15
FN1
GPNMB
SLC39A6
TPBG
GPR20
KIT
FAP
CD248
PTK7
ROR1
PDGFRA
ROR2
ALCAM
AXL
FOLH1
CD276
B3GALT5
DLL3
ADAM9
LRRC15
FN1
GPNMB
SLC39A6
TPBG
GPR20
KIT
FAP
CD248
PTK7
ROR1
PDGFRA
ROR2
ALCAM
AXL
FOLH1
CD276
MRC2
*
*
*
*
*
*
*
*
*
*
*
*
*
*
*
*
*
*
*
*
*
*
*
*
*
*
*
MRC2
E2Fregulon
*
TLS
*
*
*
*
*
HRD score
*
*
*
*
*
KIT
FN1
FAP
AXL
DLL3
PTK7
TPBG
MRC2
ROR1
ROR2
CD248
CD276
FOLH1
VTCN1
GPR20
ADAM9
ALCAM
GPNMB
LRRC15
PDGFRA
SLC39A6
B3GALT5
TFRC
CD276
PDGFRA
TPBG
FAP
LRRC15
CD248
MRC2
LGR4
EFNA4
PTK7
CD70
GPNMB
ROR2
ADAM9
SLC39A6
FN1
MET
ROR1
AXL
EGFR
*
*
*
*
*
*
*
*
*
*
*
*
*
*
CD276
PDGFRA
TPBG
FAP
LRRC15
CD248
MRC2
LGR4
EFNA4
PTK7
CD70
GPNMB
ROR2
ADAM9
SLC39A6
FN1
MET
ROR1
AXL
EGFR
E2Fregulon
*
TLS
HRD score
AXL
FN1
FAP
MET
PTK7
CD70
ROR2
ROR1
LGR4
TFRC
TPBG
EGFR
MRC2
CD276
CD248
EFNA4
ADAM9
LRRC15
GPNMB
PDGFRA
SLC39A6
Pearson r
-1
0
1
