# Supplementary material for: Expression of antibody–drug conjugate targets in soft tissue sarcomas
Source: ESMO Open. 2025 Oct 4;10(10):105837. doi: 10.1016/j.esmoop.2025.105837 (PMC12528890; doi:10.1016/j.esmoop.2025.105837)
Supplement: Supplementary Figure 4 [file mmc4.pptx]

## Slide 1
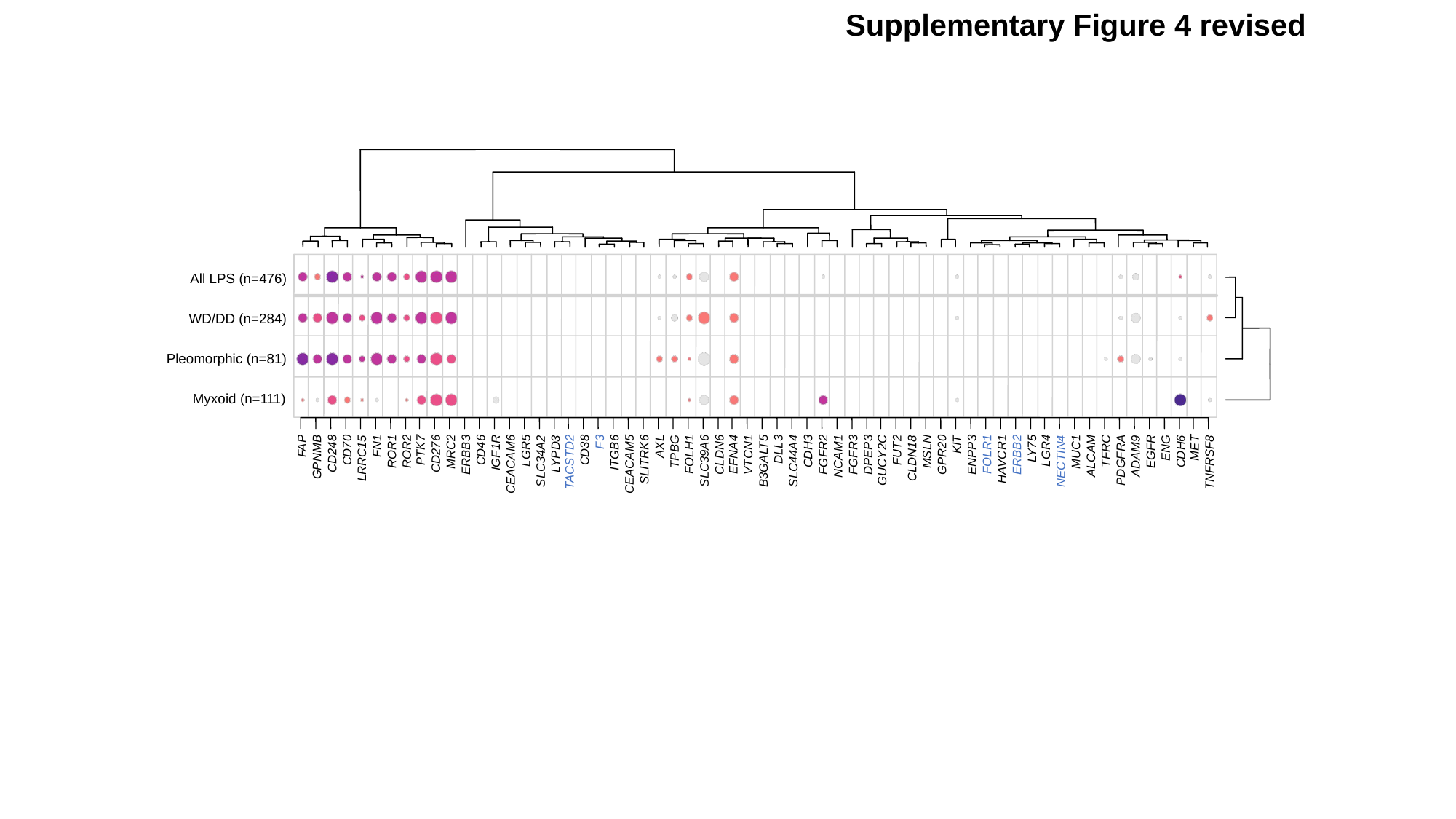

Supplementary Figure 4 revised
All LPS (n=476)
WD/DD (n=284)
Pleomorphic (n=81)
Myxoid (n=111)
FAP
GPNMB
CD248
CD70
LRRC15
FN1
ROR1
ROR2
PTK7
CD276
MRC2
ERBB3
CD46
IGF1R
CEACAM6
LGR5
SLC34A2
LYPD3
TACSTD2
CD38
F3
ITGB6
CEACAM5
SLITRK6
AXL
TPBG
FOLH1
SLC39A6
CLDN6
EFNA4
VTCN1
B3GALT5
DLL3
SLC44A4
CDH3
FGFR2
NCAM1
FGFR3
DPEP3
GUCY2C
FUT2
CLDN18
MSLN
GPR20
KIT
ENPP3
FOLR1
HAVCR1
ERBB2
LY75
LGR4
NECTIN4
MUC1
ALCAM
TFRC
PDGFRA
ADAM9
EGFR
ENG
CDH6
MET
TNFRSF8
