# Supplementary material for: Expression of antibody–drug conjugate targets in soft tissue sarcomas
Source: ESMO Open. 2025 Oct 4;10(10):105837. doi: 10.1016/j.esmoop.2025.105837 (PMC12528890; doi:10.1016/j.esmoop.2025.105837)
Supplement: Supplementary Figures and Tables Legends [file mmc14.docx]

**Supplementary Files**

**Supplementary Table 1: List of sarcoma and normal samples data sets included in our analysis.**

**Supplementary Methods: Gene expression data normalization and accuracy**

**Supplementary Table 2: List of ADC target genes and mRNA/protein expression correlation in DepMap (21Q4).**

**Supplementary Table 3: List of ADC sensitivity genes and mRNA/protein expression correlation in DepMap (21Q4).**

**Supplementary Figure 1: Hierarchical clustering of 1,664. STS samples and 62 ADC target genes.**

Each column represents one sample and each line represent one target gene. The expression level of each gene in a single sample is relative to its median abundance across the 1,664 STS samples and is depicted according to the color scale (bottom). Red and green, expression levels above and below the median, respectively. The magnitude of deviation from the median is represented by the color saturation. The dendrograms of samples and genes represent overall similarities in gene expression profiles. Bottom: the pathological STS type of samples is represented according to the color ladder (bottom right).

**Supplementary Table 4: Percentage of STS samples with ADC target expression superior to the 80^th^ percentile of expression in normal samples and tumor/normal tissue FC.**

**Supplementary Table 5: Number of ADC targets overexpressed in at least 75%, 50%, 25%, and 10% of samples.**

**Supplementary Table 6: Prognostic univariate and multivariate analyses for DFS of ADC target expression in STS types.**

**Supplementary Figure 2: Co-expression of ADC targets and signatures of therapeutic vulnerability in UPS and SVS.**

Pair-wise correlation matrix of ADC targets expression based on the Pearson’ correlation (r) in the UPS type (**A**) and the SVS type (**B**). The correlation is color-coded as indicated by the scale: red, positive correlation; blue, negative correlation; and darker colors indicate higher correlations. The significant correlations (one-sided Pearson’s correlation coefficients (greater alternative) with p-values adjusted for multiple comparisons using Bonferroni’s correction) are indicated by a white star (q<0.05).

**Supplementary Figure 3: Co-expression of ADC targets and signatures of therapeutic vulnerability in MFS and GIST.**

Pair-wise correlation matrix of ADC targets expression based on the Pearson’ correlation (r) in the MFS type (**A**) and the GIST type (**B**). The correlation is color-coded as indicated by the scale: red, positive correlation; blue, negative correlation; and darker colors indicate higher correlations. The significant correlations (one-sided Pearson’s correlation coefficients (greater alternative) with p-values adjusted for multiple comparisons using Bonferroni’s correction) are indicated by a white star (q<0.05).

**Supplementary Table 7: Pair-wise correlation matrix of ADC targets and signatures of therapeutic vulnerability in all STS types.**

In each matrix, the values under the diagonal line represent the r Pearson correlation, the values above the diagonal line represent the Bonferroni-corrected q-values.

**Supplementary Table 8: Percentage of STS samples with expression of ADC sensitivity/resistance genes superior to the 80^th^ percentile of expression in normal samples and tumor/normal tissue FC.**

**Supplementary Figure 4: Expression of ADC targets in LPS subtypes and normal tissue samples.**

**A/** Expression of 62 ADC targets in all LPS and in each LPS subtype compared to expression in all 7,414 normal tissue samples. Each column represents one gene and each line represent one LPS subtype. The dot size represents the percentage of LPS samples in each subtype with expression superior to the 80^th^ percentile of expression in normal samples, and the color code represents the tumor/normal tissue expression FC, as indicated in the scales (right).

**Supplementary Table 9: Positivity rates according to mRNA and protein expression for four ADC targets in STS.**
