# Supplementary material for: Expression of antibody–drug conjugate targets in soft tissue sarcomas
Source: ESMO Open. 2025 Oct 4;10(10):105837. doi: 10.1016/j.esmoop.2025.105837 (PMC12528890; doi:10.1016/j.esmoop.2025.105837)
Supplement: Supplementary Methods Revised [file mmc15.docx]

*ESMO Open – Research Article -* *ESMOOP-D-24-01246 Revised*

**Expression of antibody-drug conjugate targets in soft tissue sarcomas**

François Bertucci, Pascal FINETTI, Léna MESCAM,

Audrey MONNEUR, Alix FREJAFON, Axel LE CESNE, Isabelle TREILLEUX

Antoine ITALIANO, Mehdi BRAHMI, Jean-Yves BLAY, Emilie MAMESSIER^1^

**Supplementary Methods: Gene expression data normalization and accuracy**

The pre-analytic processing first included normalization of each data set separately, by using Robust Multichip Average[^36^](#_ENREF_36) with the non-parametric quantile algorithm for the raw Affymetrix data and quantile normalization for the available processed non-Affymetrix microarray data. Normalization was done in R using Bioconductor and associated packages. Then, we mapped hybridization probes across the different technological platforms as reported.[^37^](#_ENREF_37) When multiple probes mapped to the same GeneID, we retained the one with the highest variance in each data set. We log_2_-transformed the already normalized RNAseq data from the UCSC Xena transcriptome database, which includes the TCGA tumor samples and the normal samples of the GTEx project. Next, the batch effects were corrected across the 26 studies using standardization on the 122 selected genes of interest (62 ADC targets and 60 response/resistance genes) and 150 genes used as controls for data batch normalization.

The selection of these 150 control genes comprised the following steps shown in the Figure 1 below. First, we applied hierarchical clustering to two of the 26 data sets (TCGA set and Yang et al‘s set) that included all six STS types of interest. Second, we selected 6 biologically relevant gene clusters (GIST, muscle, lipid metabolism, proliferation, immune, and early response), representing 150 genes. The full list of genes is at the end of this file.

Figure 1

Each study was heterogeneous, encompassing different types of STS. Such batch normalization was performed in a stepwise manner, using LMS samples from TCGA as initial reference for average and standard deviation. For datasets that did not include LMS, standardization was performed using a previously normalized dataset with the highest proportion of the same predominant non-LMS type as reference. Such batch normalization was then applied to the 150 control genes and the 122 genes of interest.

The accuracy of batch normalization was then checked as follows. Supplementary analyses, including hierarchical clustering and Principal Component Analysis (PCA), were applied to the pre-batch normalization *versus* post-batch normalization data to show that the batch effects had been effectively addressed and that the results provided were truly associated with the biology of the sarcomas under study as opposed to technical variation or batch effects associated with different datasets from different institutions/consortia.

First, we applied hierarchical clustering to the pre-batch normalized and to the batch normalized mRNA expression levels of 150 control genes in our 1,664 tumor samples, and then compared the biological coherence of results between the two types of data. As shown in the Figure 2 below, i) the robust gene clusters identified using the *pvclust* algorithm (p<5%: see the gene dendrograms) were rare (4 clusters) and small (low number of genes) with the pre-batch normalized data, whereas they were more numerous (6 clusters) and larger (higher number of genes) with the batch normalized data; ii) the six biologically relevant gene clusters were complete and not fragmented with the batch-normalized data, whereas they were fragmented with the pre-batch normalized data; iii) the confrontation of gene clusters with the pathological type of tumor samples and the tumor grade revealed the expected strong biologically relevant correlations with the batch-normalized data, which were much less observed with the pre-batch normalized data.

Figure 2

This last point was also evidenced in the Figure 3 below, showing the expected biologically relevant correlations between the metagene of gene clusters and pathological sarcoma type (higher expression of the GIST gene cluster in the GIST samples *versus* other STS types, higher expression of the muscle gene cluster in the LMS samples *versus* other tumor types, higher expression of the lipid metabolism gene cluster in the LPS samples *versus* other tumor types), and between the metagene of the proliferation gene cluster and the pathological STS grade (higher expression grade 3 samples *versus* grade 1-2 samples). For each gene cluster, the correlations were much stronger with the batch normalized data than with the pre-batch normalized data.

Figure 3

Second, the performance of the batch normalization in removing the differences in gene expression between the 26 different data sets based on the use of different technological platforms included in the study, was also assessed using Principal Component Analysis (PCA) on the raw and batch-normalized data of the 1,664 samples and the 122 genes of interest. As shown in the Figure 4 below, prior to the batch-normalization (top), the samples in the 2D scatter plot representation were very scattered and clearly grouped according to the data set of origin (left: each color represents a set), leading to a large scattering of tumor samples of a given pathological type. By contrast, after batch-normalization (bottom), the samples and the data sets of origin were much less scattered (left), and the tumor samples of a given pathological type were much more grouped together, suggesting that the inter-batch technical differences have been removed by the batch-normalization.

****Figure 4

| **List of 150 control genes used for the batch normalization** | |  |  |  |
| --- | --- | --- | --- | --- |
|  |  |  |  |  |
| **Symbol** | **Description** | **map_location** | **Entrez Gene ID** | **Gene cluster** |
| DOK6 | docking protein 6 | 18q22.2 | 220164 | GIST |
| ATP1B1 | ATPase Na+/K+ transporting subunit beta 1 | 1q24.2 | 481 | GIST |
| CHMP3 | charged multivesicular body protein 3 | 2p11.2 | 51652 | GIST |
| PFKM | phosphofructokinase, muscle | 12q13.11 | 5213 | GIST |
| ZDHHC17 | zinc finger DHHC-type palmitoyltransferase 17 | 12q21.2 | 23390 | GIST |
| BBX | BBX high mobility group box domain containing | 3q13.12 | 56987 | GIST |
| ACSL3 | acyl-CoA synthetase long chain family member 3 | 2q36.1 | 2181 | GIST |
| CD164 | CD164 molecule | 6q21 | 8763 | GIST |
| CUL5 | cullin 5 | 11q22.3 | 8065 | GIST |
| SLC12A2 | solute carrier family 12 member 2 | 5q23.3 | 6558 | GIST |
| PDE2A | phosphodiesterase 2A | 11q13.4 | 5138 | GIST |
| SPRY4 | sprouty RTK signaling antagonist 4 | 5q31.3 | 81848 | GIST |
| BTBD3 | BTB domain containing 3 | 20p12.2 | 22903 | GIST |
| GFOD1 | Gfo/Idh/MocA-like oxidoreductase domain containing 1 | 6p24.1-p23 | 54438 | GIST |
| GLP2R | glucagon like peptide 2 receptor | 17p13.1 | 9340 | GIST |
| AMPD3 | adenosine monophosphate deaminase 3 | 11p15.4 | 272 | GIST |
| ETV1 | ETS variant transcription factor 1 | 7p21.2 | 2115 | GIST |
| DPP10 | dipeptidyl peptidase like 10 | 2q14.1 | 57628 | GIST |
| ANO1 | anoctamin 1 | 11q13.3 | 55107 | GIST |
| ANXA3 | annexin A3 | 4q21.21 | 306 | GIST |
| PRKCQ | protein kinase C theta | 10p15.1 | 5588 | GIST |
| KIT | KIT proto-oncogene, receptor tyrosine kinase | 4q12 | 3815 | GIST |
| ANKS1A | ankyrin repeat and sterile alpha motif domain containing 1A | 6p21.31 | 23294 | GIST |
| PLAGL1 | PLAG1 like zinc finger 1 | 6q24.2 | 5325 | GIST |
| NBL1 | NBL1, DAN family BMP antagonist | 1p36.13 | 4681 | GIST |
| TMEM204 | transmembrane protein 204 | 16p13.3 | 79652 | GIST |
| NPR3 | natriuretic peptide receptor 3 | 5p13.3 | 4883 | GIST |
| AHCYL2 | adenosylhomocysteinase like 2 | 7q32.1 | 23382 | GIST |
| NBN | nibrin | 8q21.3 | 4683 | GIST |
| KIF20A | kinesin family member 20A | 5q31.2 | 10112 | Prolif |
| DEPDC1 | DEP domain containing 1 | 1p31.3 | 55635 | Prolif |
| BUB1 | BUB1 mitotic checkpoint serine/threonine kinase | 2q13 | 699 | Prolif |
| CKAP2L | cytoskeleton associated protein 2 like | 2q14.1 | 150468 | Prolif |
| ASPM | assembly factor for spindle microtubules | 1q31.3 | 259266 | Prolif |
| CENPF | centromere protein F | 1q41 | 1063 | Prolif |
| KIF14 | kinesin family member 14 | 1q32.1 | 9928 | Prolif |
| BUB1B | BUB1 mitotic checkpoint serine/threonine kinase B | 15q15.1 | 701 | Prolif |
| NUSAP1 | nucleolar and spindle associated protein 1 | 15q15.1 | 51203 | Prolif |
| TOP2A | DNA topoisomerase II alpha | 17q21.2 | 7153 | Prolif |
| CDCA8 | cell division cycle associated 8 | 1p34.3 | 55143 | Prolif |
| KIF18B | kinesin family member 18B | 17q21.31 | 146909 | Prolif |
| KIF4A | kinesin family member 4A | Xq13.1 | 24137 | Prolif |
| TPX2 | TPX2 microtubule nucleation factor | 20q11.21 | 22974 | Prolif |
| SGO1 | shugoshin 1 | 3p24.3 | 151648 | Prolif |
| KIF23 | kinesin family member 23 | 15q23 | 9493 | Prolif |
| PRC1 | protein regulator of cytokinesis 1 | 15q26.1 | 9055 | Prolif |
| NEK2 | NIMA related kinase 2 | 1q32.3 | 4751 | Prolif |
| STIL | STIL centriolar assembly protein | 1p33 | 6491 | Prolif |
| TTK | TTK protein kinase | 6q14.1 | 7272 | Prolif |
| KIF11 | kinesin family member 11 | 10q23.33 | 3832 | Prolif |
| MKI67 | marker of proliferation Ki-67 | 10q26.2 | 4288 | Prolif |
| SPAG5 | sperm associated antigen 5 | 17q11.2 | 10615 | Prolif |
| NUF2 | NUF2 component of NDC80 kinetochore complex | 1q23.3 | 83540 | Prolif |
| NCAPG | non-SMC condensin I complex subunit G | 4p15.31 | 64151 | Prolif |
| EGR2 | early growth response 2 | 10q21.3 | 1959 | Early Response |
| EGR3 | early growth response 3 | 8p21.3 | 1960 | Early Response |
| EGR1 | early growth response 1 | 5q31.2 | 1958 | Early Response |
| FOSB | FosB proto-oncogene, AP-1 transcription factor subunit | 19q13.32 | 2354 | Early Response |
| FOS | Fos proto-oncogene, AP-1 transcription factor subunit | 14q24.3 | 2353 | Early Response |
| NR4A1 | nuclear receptor subfamily 4 group A member 1 | 12q13.13 | 3164 | Early Response |
| C16orf54 | chromosome 16 open reading frame 54 | 16p11.2 | 283897 | Immune |
| ADGRG5 | adhesion G protein-coupled receptor G5 | 16q21 | 221188 | Immune |
| CD8B | CD8 subunit beta | 2p11.2 | 926 | Immune |
| GZMH | granzyme H | 14q12 | 2999 | Immune |
| ICOS | inducible T cell costimulator | 2q33.2 | 29851 | Immune |
| CD48 | CD48 molecule | 1q23.3 | 962 | Immune |
| FASLG | Fas ligand | 1q24.3 | 356 | Immune |
| CD8A | CD8 subunit alpha | 2p11.2 | 925 | Immune |
| CD96 | CD96 molecule | 3q13.13-q13.2 | 10225 | Immune |
| GZMK | granzyme K | 5q11.2 | 3003 | Immune |
| CCL5 | C-C motif chemokine ligand 5 | 17q12 | 6352 | Immune |
| CD27 | CD27 molecule | 12p13.31 | 939 | Immune |
| CD52 | CD52 molecule | 1p36.11 | 1043 | Immune |
| CD3G | CD3 gamma subunit of T-cell receptor complex | 11q23.3 | 917 | Immune |
| CXCR6 | C-X-C motif chemokine receptor 6 | 3p21.31 | 10663 | Immune |
| CD247 | CD247 molecule | 1q24.2 | 919 | Immune |
| CD5 | CD5 molecule | 11q12.2 | 921 | Immune |
| CXCR3 | C-X-C motif chemokine receptor 3 | Xq13.1 | 2833 | Immune |
| CD2 | CD2 molecule | 1p13.1 | 914 | Immune |
| CD3E | CD3 epsilon subunit of T-cell receptor complex | 11q23.3 | 916 | Immune |
| CD3D | CD3 delta subunit of T-cell receptor complex | 11q23.3 | 915 | Immune |
| LCK | LCK proto-oncogene, Src family tyrosine kinase | 1p35.2 | 3932 | Immune |
| IL2RG | interleukin 2 receptor subunit gamma | Xq13.1 | 3561 | Immune |
| SIRPG | signal regulatory protein gamma | 20p13 | 55423 | Immune |
| SLA2 | Src like adaptor 2 | 20q11.23 | 84174 | Immune |
| UBASH3A | ubiquitin associated and SH3 domain containing A | 21q22.3 | 53347 | Immune |
| ITGAL | integrin subunit alpha L | 16p11.2 | 3683 | Immune |
| TIGIT | T cell immunoreceptor with Ig and ITIM domains | 3q13.31 | 201633 | Immune |
| SIT1 | signaling threshold regulating transmembrane adaptor 1 | 9p13.3 | 27240 | Immune |
| SLAMF6 | SLAM family member 6 | 1q23.2-q23.3 | 114836 | Immune |
| ACAP1 | ArfGAP with coiled-coil, ankyrin repeat and PH domains 1 | 17p13.1 | 9744 | Immune |
| TBC1D10C | TBC1 domain family member 10C | 11q13.2 | 374403 | Immune |
| PTPRCAP | protein tyrosine phosphatase receptor type C associated protein | 11q13.2 | 5790 | Immune |
| MAP4K1 | mitogen-activated protein kinase kinase kinase kinase 1 | 19q13.2 | 11184 | Immune |
| TRAT1 | T cell receptor associated transmembrane adaptor 1 | 3q13.13 | 50852 | Immune |
| ZAP70 | zeta chain of T cell receptor associated protein kinase 70 | 2q11.2 | 7535 | Immune |
| TBX21 | T-box transcription factor 21 | 17q21.32 | 30009 | Immune |
| TRAF3IP3 | TRAF3 interacting protein 3 | 1q32.2 | 80342 | Immune |
| ZNF831 | zinc finger protein 831 | 20q13.32 | 128611 | Immune |
| THEMIS | thymocyte selection associated | 6q22.33 | 387357 | Immune |
| SPN | sialophorin | 16p11.2 | 6693 | Immune |
| NKG7 | natural killer cell granule protein 7 | 19q13.41 | 4818 | Immune |
| PYHIN1 | pyrin and HIN domain family member 1 | 1q23.1 | 149628 | Immune |
| SLAMF7 | SLAM family member 7 | 1q23.3 | 57823 | Immune |
| LY9 | lymphocyte antigen 9 | 1q23.3 | 4063 | Immune |
| HSH2D | hematopoietic SH2 domain containing | 19p13.11 | 84941 | Immune |
| ZBP1 | Z-DNA binding protein 1 | 20q13.31 | 81030 | Immune |
| LTB | lymphotoxin beta | 6p21.33 | 4050 | Immune |
| S1PR4 | sphingosine-1-phosphate receptor 4 | 19p13.3 | 8698 | Immune |
| SLAMF1 | signaling lymphocytic activation molecule family member 1 | 1q23.3 | 6504 | Immune |
| CTDSP2 | CTD small phosphatase 2 | 12q14.1 | 10106 | Lipid metabolism |
| EEF1AKMT3 | EEF1A lysine methyltransferase 3 | 12q14.1 | 25895 | Lipid metabolism |
| CDK4 | cyclin dependent kinase 4 | 12q14.1 | 1019 | Lipid metabolism |
| METTL1 | methyltransferase 1, tRNA methylguanosine | 12q14.1 | 4234 | Lipid metabolism |
| TSFM | Ts translation elongation factor, mitochondrial | 12q14.1 | 10102 | Lipid metabolism |
| TSPAN31 | tetraspanin 31 | 12q14.1 | 6302 | Lipid metabolism |
| MDM2 | MDM2 proto-oncogene | 12q15 | 4193 | Lipid metabolism |
| AKAP1 | A-kinase anchoring protein 1 | 17q22 | 8165 | Muscle |
| ACTA2 | actin alpha 2, smooth muscle | 10q23.31 | 59 | Muscle |
| ASB2 | ankyrin repeat and SOCS box containing 2 | 14q32.12 | 51676 | Muscle |
| DSTN | destrin, actin depolymerizing factor | 20p12.1 | 11034 | Muscle |
| JPH2 | junctophilin 2 | 20q13.12 | 57158 | Muscle |
| ITGB1BP2 | integrin subunit beta 1 binding protein 2 | Xq13.1 | 26548 | Muscle |
| ALDH1B1 | aldehyde dehydrogenase 1 family member B1 | 9p13.1 | 219 | Muscle |
| ACTG2 | actin gamma 2, smooth muscle | 2p13.1 | 72 | Muscle |
| CNN1 | calponin 1 | 19p13.2 | 1264 | Muscle |
| KCNQ4 | potassium voltage-gated channel subfamily Q member 4 | 1p34.2 | 9132 | Muscle |
| FBXL22 | F-box and leucine rich repeat protein 22 | 15q22.31 | 283807 | Muscle |
| CSRP1 | cysteine and glycine rich protein 1 | 1q32.1 | 1465 | Muscle |
| LMOD1 | leiomodin 1 | 1q32.1 | 25802 | Muscle |
| KCNMB1 | potassium calcium-activated channel subfamily M regulatory beta subunit 1 | 5q35.1 | 3779 | Muscle |
| MYH11 | myosin heavy chain 11 | 16p13.11 | 4629 | Muscle |
| PPP1R12B | protein phosphatase 1 regulatory subunit 12B | 1q32.1 | 4660 | Muscle |
| IRAG1 | inositol 1,4,5-triphosphate receptor associated 1 | 11p15.4 | 10335 | Muscle |
| MYOCD | myocardin | 17p12 | 93649 | Muscle |
| MYLK | myosin light chain kinase | 3q21.1 | 4638 | Muscle |
| SLMAP | sarcolemma associated protein | 3p14.3 | 7871 | Muscle |
| SORBS1 | sorbin and SH3 domain containing 1 | 10q24.1 | 10580 | Muscle |
| NPAS4 | neuronal PAS domain protein 4 | 11q13.2 | 266743 | Muscle |
| RPE65 | retinoid isomerohydrolase RPE65 | 1p31.3 | 6121 | Muscle |
| RASL12 | RAS like family 12 | 15q22.31 | 51285 | Muscle |
| LMO1 | LIM domain only 1 | 11p15.4 | 4004 | Muscle |
| ROPN1B | rhophilin associated tail protein 1B | 3q21.2 | 152015 | Muscle |
| ROPN1 | rhophilin associated tail protein 1 | 3q21.1 | 54763 | Muscle |
| MOG | myelin oligodendrocyte glycoprotein | 6p22.1 | 4340 | Muscle |
| MYL9 | myosin light chain 9 | 20q11.23 | 10398 | Muscle |
| TAGLN | transgelin | 11q23.3 | 6876 | Muscle |
| TSPAN2 | tetraspanin 2 | 1p13.2 | 10100 | Muscle |
| SYNPO2 | synaptopodin 2 | 4q26 | 171024 | Muscle |
| SYNM | synemin | 15q26.3 | 23336 | Muscle |
